# Crucifixion and median neuropathy

**DOI:** 10.1002/brb3.132

**Published:** 2013-03-18

**Authors:** Jacqueline M Regan, Kiarash Shahlaie, Joseph C Watson

**Affiliations:** 1 Department of Neurosciences, Inova Fairfax HospitalFalls Church, Virgina, 22042; 2 Department of Neurological Surgery, University of CaliforniaDavis, Sacramento, California, 95817

**Keywords:** Benediction sign, crucifixion, median nerve, neuropathy

## Abstract

Crucifixion as a means of torture and execution was first developed in the 6th century B.C. and remained popular for over 1000 years. Details of the practice, which claimed hundreds of thousands of lives, have intrigued scholars as historical records and archaeological findings from the era are limited. As a result, various aspects of crucifixion, including the type of crosses used, methods of securing victims to crosses, the length of time victims survived on the cross, and the exact mechanisms of death, remain topics of debate. One aspect of crucifixion not previously explored in detail is the characteristic hand posture often depicted in artistic renditions of crucifixion. In this posture, the hand is clenched in a peculiar and characteristic fashion: there is complete failure of flexion of the thumb and index finger with partial failure of flexion of the middle finger. Such a “crucified clench” is depicted across different cultures and from different eras. A review of crucifixion history and techniques, median nerve anatomy and function, and the historical artistic depiction of crucifixion was performed to support the hypothesis that the “crucified clench” results from proximal median neuropathy due to positioning on the cross, rather than from direct trauma of impalement of the hand or wrist.

“Of all punishments, it is the most cruel and most terrifying.”

― Cicero (Roman Statesman), 1st century B.C. (LeBec 1925)

## Brief History of Crucifixion

Crucifixion as a means of state-sponsored torture and execution likely began in the Persian Empire five centuries before the birth of Christ. It was originally “designed” as a means of executing condemned criminals without allowing their feet to touch “holy ground” (Jackson 1909; Barbet 1953; Tenney 1964). This practice spread rapidly throughout the Persian Empire (Friedrich 1971; Shrier 2002), and was adopted by nearby Indian, Scythian, Taurian, and Assyrian societies (Holoubek and Holoubek 1995).

In the 4th century B.C., Alexander the Great adopted crucifixion from the Persians, introducing it to Egypt, Carthage, and the Roman Empire. In Rome, the practice rapidly flourished, evolving into a brutal means of executing revolutionaries, slaves, and foreign criminals (Roman citizens were protected from the torture except in cases of deserting soldiers) (Depasquale and Burch 1963; Hengel 1977). In the centuries that followed, many mass crucifixions were performed in the Roman Empire, often adjacent to heavily traveled passageways to serve as warnings to foreigners and potential invaders (Edwards et al. 1986; Hoare 1994).

In its earliest Persian form, the condemned were tied with rope or impaled to an upright post or tree and left to die. In Rome, however, crucifixion developed into a lengthy, torturous ceremony (Edwards et al. 1986). The condemned were initially stripped of their clothing, tied to a pole, and publicly ridiculed while flogged with a flagrum consisting of leather bands attached to metal balls or small bones (Holoubek and Holoubek 1995). After the flogging, the victim was forced to carry a 75–125 pound patibulum across his shoulders to the site of crucifixion, typically located outside the city walls in view of travelers-by (Barbet 1953; Edwards et al. 1986; Hoare 1994; Holoubek and Holoubek 1995). Suffering from significant blood loss and physical exhaustion, the condemned was then offered a mild analgesic drink of wine and myrrh and thrown back upon the patibulum to be secured. Various types of crosses were developed in Rome for this practice, including the “T-shaped” tau and “┼-shaped” Latin crosses (Barbet 1953; Davis 1965, 1976; Hengel 1977; Lumpkin 1978; Edwards et al. 1986). For either style, the distal upper and lower extremities were typically secured using large iron spikes (rope was used in areas where metals were scarce) (Barbet 1953; Tzaferis 1971; Zias and Sekeles 1985; Edwards et al. 1986; Holoubek and Holoubek 1995).

Various factors, including the severity of flogging wounds, dehydration, weather conditions, type of cross used, and the condemned man's age, determined the length of time victims typically survived on the cross (Barbet 1953; Hoare 1994; Holoubek and Holoubek 1995). Most victims died within 24 to 36 h, at which point guards delivered a blow to the right chest and heart (Edwards et al. 1986; Holoubek and Holoubek 1995). If the condemned was punctured postmortem the fluid would flood out of the wound, while if stabbed antemortem, before blood and pulmonary edema saturated the lungs, no liquid would drain. This was an efficient and effective way to confirm death of those being crucified.

## Controversial Aspects of Crucifixion

Various aspects of crucifixion are not fully understood, and have therefore generated significant scholarly interest. For example, some authors propose that death by crucifixion results from asphyxiation (Barbet 1953; Bucklin 1963; Depasquale and Burch 1963; Davis 1965; Lumpkin 1978) while others have implicated cardiac rupture (Stroud 1871; Whitaker 1935; Bergsma 1948) or shock (Tenney 1964; Zugibe 1989; Holoubek and Holoubek 1995). In 1989, the Canadian pathologist Zugibe explored this topic experimentally, monitoring young male volunteers strapped to crosses for prolonged periods of time. He found no evidence of respiratory or cardiac compromise, lending support to the “shock theory.”

**Figure 1:**
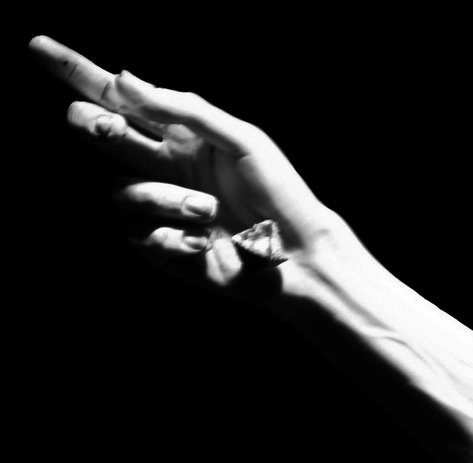
Image showing the crucified clench hand position with nail.

Techniques used to secure the upper extremities to the cross have also been explored by scholars of crucifixion. Popular belief and many artistic depictions have long depicted nails passing through the palms of the hands of the crucified victim (Fig. 1). Many critics have challenged this theory, however, citing the mechanical inability of the hands to support the weight of the crucified body on the cross (Barbet 1953; Haas 1970; Tzaferis 1971; Davis 1976; Weaver 1980; Edwards et al. 1986). Cadaveric studies have indeed supported this criticism, demonstrating that nails simply tear through the flesh between the metacarpal bones when secured to a cross in this manner (Barbet 1953). If nails are passed through the wrist, however, the arms can support the weight of the body because of mechanical support from the transverse carpal ligament, flexor retinaculum, and carpal bones of the hand (Shrier 2002). Ossuary findings near Jerusalem and the Shroud of Turin have provided additional evidence on the topic, supporting the theory that nailing of the wrists was performed between the radius and ulna bones (Haas 1970; Tzaferis 1971; Weaver 1980).

**Figure 2:**
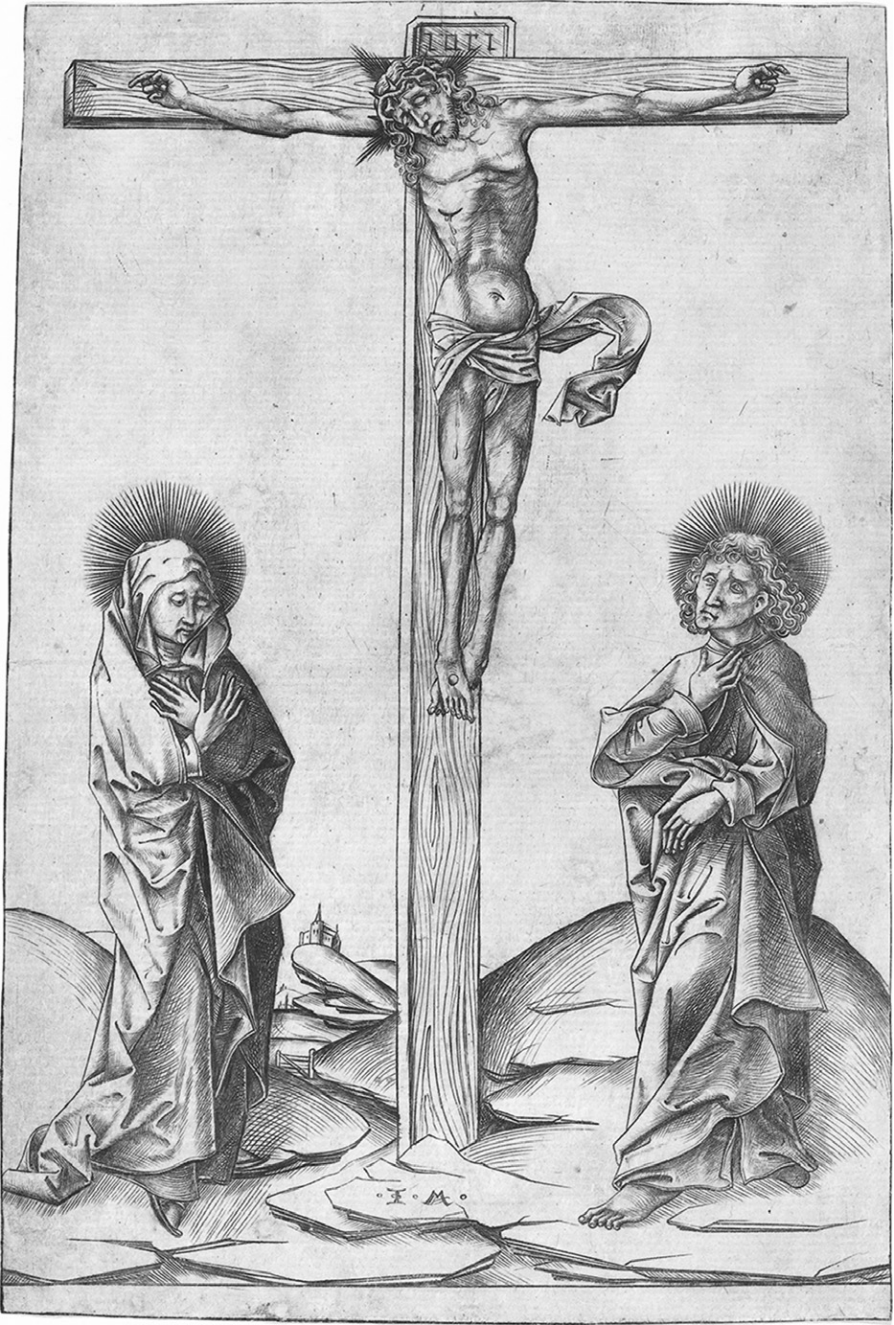
Image from the United States National Gallery of Art, Washington, D.C.; The Crucifixion, c. 1475 engraving, Israhel van Meckenem, German, c. 1445-1503. Rosenwald Collection 1943.3.103.

Many artistic depictions also show the hands in a characteristic clenched posture (Fig. 2). In this crucified clench position, the third and fourth fingers are completely flexed, the middle finger is partially flexed, and there is complete extension of the index finger and thumb. Passage of a nail through the hand or wrist, with resultant distal median nerve damage, would not result in this hand posture, as finger and thumb flexors in the forearm would be spared. This crucified clench, on the other hand, results from median nerve dysfunction at the elbow/proximal forearm, likely as a consequence of prolonged upper extremity abduction, extension, and external rotation on the cross.

Starting in the 5th century, artistic renditions of the crucifixion began to appear on ivory caskets and grew to be a popular subject of focus of all art media in the 13th century and throughout the renaissance era. In many works, the condemned was shown with the half-clutched hand position, the thumb and index finger extended, the middle only partially flexed, and the ring and little finger fully flexed. This hand position on the crucifix appears to have been first seen in art in a rendition in the late 8th to early 9th century made in Constantinople (Byzantine 8th–9th century), though earlier renditions, such as that of a 6th century reliquary casket found in Bawit (6th century), illustrate a partial crucified clench through obvious failure of flexion of thumb and index fingers. Though the crucified clench is popular in many works depicting crucifixion, the earliest versions show only straight hand position with no flexion of any fingers. Representations of crucifixions began to appear only after the practice of crucifixion was banned by Constantine I in the fourth century; however, crucifixions continued in non-Christian countries into the early 1800s (Gibson and Cohn 2007). This leads to debate of whether the crucified clench was from an invented artistic style or based on true observation.

This crucified clenched described here is also a well-known benediction sign used in the churches by priests and popes; however, the origin of this hand position and its relation to Christianity is unclear (Elworthy 1900). The extension of the thumb and first two fingers with the flexion of the ring and little fingers has been described in the late 2nd century by Apuleius in his *Metamorphoses* as the gesture of an orator, though the sign was believed to be sacred even at that time (Elworthy 1900; Apuleius et al. 1915). The benediction sign is clearly depicted in the 6th century Ravenna mosaics picturing angels, prophets, priests, and Christ himself, many of times denoting Christ's death on the cross, but rarely illustrating the act of crucifixion itself. Depicting the agonizing act of crucifixion and not just Christ with the cross may have been a stigma around the time of its use early in the first millennium and thus muted the origin of the hand position with it. Though this gesture may have been used in the past and present as a symbol of prayer, its origins as a symbol is currently undecided and may rest in the act of crucifixion. The truth that may never be known for sure is whether the hand position was first the crucified clench or the benediction sign.

From architecture in the 16th century Sé cathedral in India (Argueiros and Simao 16th–17th century), to the 6th century casket in Bawit (6th century), the crucified clench is a hand position that is noticed in crucifixion works across time and culture. Though the hand position only began to appear in crucifixion depictions in the 8th century, it flourished throughout many areas where crucifixion was previously prevalent and in non-Christian countries where the practice continued. The archive of crucifixion renditions comes primarily from the time after the practice was discontinued, and thus there would have been little if any direct observation of the hand position on the cross. However, the ubiquitous depiction of the crucified clench across time, cultures, and artistic styles suggests that true observations were made or passed down through time.

## Median Neuropathy

When secured to the cross, the victim's upper extremities are maintained in a characteristic position, with the shoulders abducted ∼135º, the glenohumeral joint externally rotated, the elbow extended, the forearm supinated, and the wrist radially deviated and extended. There is also significant traction on the upper extremities across all joints due to the weight of the suspended body. It is known from human cadaver studies that significant median nerve strain results from certain shoulder, elbow, and wrist positions. Wright et al. (1996), for example, reported significant median nerve strain and excursion at the wrist and elbow in fresh-frozen cadavers with wrist extension, radial deviation, and shoulder abduction. Kleinrensink et al. (1995) similarly used “buckle” force transducers to measure median nerve tension in cadavers, reporting significant tension with shoulder abduction, retroflexion, and external rotation – postures held during crucifixion. Byl et al. (2002) also found significant median nerve excursion at the proximal forearm with shoulder abduction, elbow and wrist extension. Postures assumed on the cross, therefore, result in significant mechanical strain on the median nerve at the elbow/proximal forearm.

Though positioning strain on the nerves themselves has proven to cause significant damage, animal studies have demonstrated a strong relationship between such degrees of mechanical strain and compromised blood flow to peripheral nerves. For example, mild sciatic nerve strain in rats reduces blood flow by 50% while more significant strains reduce perfusion up to 80% (Clark et al. 1992). Similar results have been reported in rabbit studies, demonstrating complete arrest of blood flow with moderate nerve strain (Ogata and Naito 1986). Mechanical strain on the order observed in cadaveric studies, therefore, results in moderate to severe peripheral nerve ischemia. Such degrees of prolonged ischemia compromise peripheral nerve function. For example, mild sciatic nerve strain maintained for 60 min in rats results in 70% decrease of action potential amplitude; more significant levels of sciatic nerve strain completely block function (Lundborg and Rydevik 1973; Wall et al. 1992). These degrees of ischemia result in cell edema with suppression of axonal transport and alterations in conduction characteristics (Wall et al. 1992; Tanoue et al. 1996; Coppieters et al. 2002). Mechanical strains observed in human cadaver studies, therefore, may disrupt action potential conductance in the proximal median nerve, resulting in functional denervation of specific forearm muscles.

While the hyperextension of the elbow during crucifixion results in strain on the median nerve, it releases tension from the ulnar nerve. When the arm is flexed the ulnar nerve is stretched in the cubital tunnel, but when the arm is positioned similar to that during crucifixion, the ulnar nerve is relaxed in the tunnel. This explains why we only see a median neuropathy and not an ulnar neuropathy in the crucified clench. As the ulnar nerve remains uninjured in the hanging position, flexion of the little and ring fingers remain intact and there is partial flexion of the middle finger, creating the iconic clench during crucifixion.

**Figure 3:**
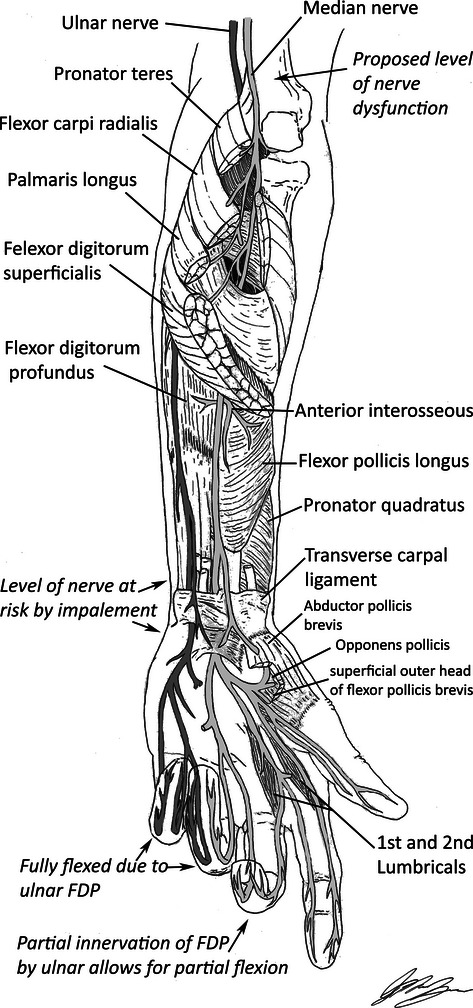
Illustration of the median and ulnar nerve anatomy. Only dysfunction of the median nerve at the elbow would result in this particular hand posture, as a result of the median involved muscles, while sparing the ulnar flexors.

The median nerve gives rise to the anterior interosseus nerve, which innervates the radial portions of the flexor digitorum profundus (flexes index and middle fingers at the distal interphalangeal joints), flexor pollicis longus (flexes phalanges of thumb), and pronator quadratus (pronates forearm). All these branches would be spared from a penetrating trauma at the wrist or palm (Fig. 3). The portion of the nerve at risk for impalement is that which innervates the abductor pollicis brevis (abducts thumb), opponens pollicis (opposition of first metacarpal), superficial outer head of the flexor pollicis brevis (flexes thumb at metacarpal-phalangeal [MCP] joint), and the first and second lumbricals (flex index and middle fingers at MCP joint). Injury here at the wrist would result in a much different hand posture than that which is depicted for crucifixion, as flexion of the thumb index and middle fingers at the MCP joints would still be possible.

Furthermore, functional denervation of target muscles results in various components of the crucified clench. Loss of pronator teres and pronator quadratus functions, for example, results in the forearm remaining extended and supinated, while loss of flexor carpi radialis and palmaris longus function maintains the hand in extension. The thumb fails to flex due to loss of flexor pollicis longus and brevis function, and cannot abduct or be drawn forward at right angles to the palm (to oppose the other digits to form a fist or clench/grasp) due to loss of abductor pollicis brevis and opponens pollicis functions. The index finger fails to flex at the distal interphalangeal joints (due to loss of flexor digitorum profundus) or proximal interphalangeal and MCP joints (due to loss of flexor digitorum superficialis and the first lumbrical). The middle finger displays a similar pattern of deficits, although these are less severe as innervation of these muscle groups (in particular the flexor digitorum profundus) is shared between median and ulnar nerve branches (the latter remain intact). This combination of deficits results in complete flexion paralysis of the index finger, partial paresis of middle finger flexion, and failure to abduct, flex, and oppose the thumb.

## Conclusion

One feature of crucifixion never before explored is the iconic clenched hand position as seen in many artistic renditions. Our hypothesis that the crucified clench resulted from a median neuropathy due to lengthy upper extremity positioning was evaluated through the exploration of crucifixion history and techniques, median nerve anatomy and function, and artistic illustrations. An experiment using volunteers would be the most conclusive way to prove this hypothesis; however, ethical considerations make this unreasonable.

Distal median nerve or even limited tendon damage could result from a nail being thrust through the hand or wrist, yet the characteristic hand positioning shown in many illustrations is diagnostic of median nerve damage at the elbow or proximal forearm; paralysis at the distal median nerve results in an entirely different hand posture with lack of thumb apposition (abduction) and lack of distal index and middle finger flexion (flexion of the fingers at the proximal [metacarpal-phalangeal] joint is spared). Through cadaver and animal studies, it has been shown that the body position while being crucified, shoulders abducted ∼135º, the glenohumeral joint externally rotated, the elbow extended, the forearm supinated, and the wrist radially deviated and extended, can cause ischemia with related significant median nerve strain at the elbow or proximal forearm. This same position releases tension on the ulnar nerve in the cubital tunnel, allowing for undisturbed flexion of the little and ring fingers in the crucified clench. The failure of flexion of the thumb and index and middle fingers that is characteristic of a median neuropathy therefore must be a result of the lengthy crucifixion ritual with its unnatural upper extremity positioning.
